# Citizenship and Learning Disabled People: The Mental Health Charity MIND’s 1970s Campaign in Historical Context

**DOI:** 10.1017/mdh.2017.55

**Published:** 2017-10

**Authors:** Jonathan Toms

**Affiliations:** Department of History, University of East Anglia, Norwich Research Park, Norwich, NR4 7TJ, UK

**Keywords:** Learning disability, Mental hygiene, Citizenship, MIND, Deinstitutionalisation

## Abstract

Current policy and practice directed towards people with learning disabilities originates in the deinstitutionalisation processes, civil rights concerns and integrationist philosophies of the 1970s and 1980s. However, historians know little about the specific contexts within which these were mobilised. Although it is rarely acknowledged in the secondary literature, MIND was prominent in campaigning for rights-based services for learning disabled people during this time. This article sets MIND’s campaign within the wider historical context of the organisation’s origins as a main institution of the inter-war mental hygiene movement. The article begins by outlining the mental hygiene movement’s original conceptualisation of ‘mental deficiency’ as the antithesis of the self-sustaining and responsible individuals that it considered the basis of citizenship and mental health. It then traces how this equation became unravelled, in part by the altered conditions under the post-war Welfare State, in part by the mental hygiene movement’s own theorising. The final section describes the reconceptualisation of citizenship that eventually emerged with the collapse of the mental hygiene movement and the emergence of MIND. It shows that representations of MIND’s rights-based campaigning (which have, in any case, focused on mental illness) as individualist, and fundamentally opposed to medicine and psychiatry, are inaccurate. In fact, MIND sought a comprehensive community-based service, integrated with the general health and welfare services and oriented around a reconstruction of learning disabled people’s citizenship rights.

Present governmental policy and professional practice towards people with learning disabilities originated in the deinstitutionalisation processes, civil rights concerns and integrationist philosophies of the 1970s and 1980s. However, historians know little about the specific contexts within which these issues were mobilised. Academic commentaries on the mental health charity MIND’s activities during this time have concentrated on its rights-based campaigning regarding mental illness. Yet, MIND was also prominent in campaigning to change government policy and professional practice for people then called mentally handicapped. There is an almost complete lack of reference to the organisation in contemporary historical overviews of these changes.^1^ Critics of MIND’s rights-based approach, (focusing on its campaigning regarding mental illness) have commonly maintained that it was individualist in nature, and fundamentally opposed to medicine and psychiatry. It is claimed that debate was framed in terms of the deprivation of liberty. This enshrined personal liberty at the cost of care and treatment.^2^ The characterisation has, in turn, been used to support the view that MIND encouraged patients’ discharge from hospitals without enough consideration of the consequences for people’s lives in the community.^3^

This article contests these representations. It argues that a proper appreciation of MIND’s rights-based campaigning requires its contextualisation with the organisation’s much longer history, which stems back, ultimately, to 1913. This history is itself intimately related to notions of citizenship. Its examination places familiar touchstones in the history of people with learning disabilities in a new light.

Under the arresting anthropological concept of ‘soul catchers’, a recent special edition of *Medical History* has examined the relationship between technologies of the mind sciences and those immaterial aspects of what, in the modern world, has become known as subjectivity.^4^ This article does not directly reflect on such relationships. Instead, it attempts the more modest task of tracing how a conjunction of the altered socio-political terrain of the post-war Welfare State, with changing approaches in the medical and allied sciences, gradually transformed conceptualisations of learning disabled people’s subjectivity and citizenship. Other recent articles in *Medical History* have reinvigorated Roy Porter’s mid-1980s clarion call for a ‘patients’ history from below’, examining in detail elements of the so-called ‘doctor-patient’ relationship and its relation to the construction and negotiation of medical knowledge in the mental health services.^5^ Again, this article does not directly address these issues. Nevertheless, the transformations in the relationship between medical services and ideas about citizenship that it examines were important precursors of the shifts in the social history of medicine during the 1980s towards unearthing and interrogating ‘the patients’ narrative’.

The first two sections of this article are devoted to an examination of MIND’s origins in the voluntary organisations of the inter-war mental hygiene movement, and its emergence as the leading body of the movement in the post-war years. They trace the intimate connections between the mental hygiene movement’s policies towards people then termed ‘mentally deficient’ and notions of citizenship. The third section examines MIND’s transformation into a rights-based campaigning organisation and the associated reconstruction of the relationship of citizenship and learning disabled people.

## 1 The Inter-war Mental Hygiene Movement

MIND’s official title was (and remains) the National Association for Mental Health (NAMH). Formed in 1946, it was an amalgamation of three leading voluntary organisations that had founded the British mental hygiene movement.^6^ These were: the Central Association for Mental Welfare (CAMW), the National Council for Mental Hygiene (NCMH) and the Child Guidance Council (CGC). The movement’s aim was to encourage the prevention, or early ascertainment and treatment, of mental disorders within the community. It concerned itself with mental illness and, what was then called, mental deficiency. Psychiatrists were highly influential but the movement also encompassed and promoted what were considered ancillary professions such as psychiatric social workers, educational psychologists and educationists.

The earliest organisation, the CAMW, had been founded in 1913 in direct response to the Mental Deficiency Act of that year.^7^ Its leading members comprised doctors and other campaigners who had been involved in pressing for this legislation. People considered ‘mentally deficient’ were considered a ‘social problem’ requiring control as well as care. Under the 1913 Act, all county and county borough authorities in England and Wales were to ascertain the number of people deemed ‘mentally defective’ and arrange institutional provision or community supervision.^8^ Doctors received a key role in diagnosis and certification procedures. Local voluntary organisations could also appoint themselves to identify and supervise people. The CAMW set itself up as the central training and coordinating body for these organisations.

Historians have commonly attributed the construction of ‘mental deficiency’ as a major social problem to eugenic concerns about national fitness.^9^ But Mathew Thomson has situated it within the wider political context of ‘adjusting to democracy’, convincingly showing an intimate connection with redefining rights and citizenship.^10^ The franchise was greatly widened between 1867 and 1918, despite continuing limitations (notably the exclusion of women under the age of thirty). This increased anxieties among some about the requirements of responsible citizenship. The ‘social problem’ of ‘mental deficiency’ became a focus for these concerns. As Thomson puts it, ‘The category of mental deficiency provided a way to conceptualise a group within the population who were non-citizens, not on grounds of wealth or class but because of an innate deficiency and social inefficiency.’^11^

The CAMW’s association of mental deficiency with ‘social inefficiency’ confronted it with people on the so-called ‘borderline’ between apparent pathology and mental health.^12^ Through this, the CAMW combined with the NCMH, the CGC and Tavistock Clinic in the inter-war formation of a movement for mental hygiene. This movement continued to campaign on the ‘problem of mental deficiency’ but it focused particularly on what it considered psychological causes of ‘social failure’ in the wider population. This focus was strongly informed by psychodynamic thinking. An emphasis on ‘emotional adjustment’ in the interests of adequate citizenship and social efficiency developed – with this construed in terms of mental health. This entailed, in particular, sensitive attention to relationships of nurture and authority in childhood upbringing.^13^ Mentally deficient children’s emotional lives and behaviour difficulties were, however, largely considered mere consequences of intellectual incapacity.

The mental hygiene movement pressed for greater institutional provision for mental defectives as a means of social and mental hygiene. But there was growing recognition that relying solely on institutionalisation was not economically viable. Instead, an integrated system of institutions and community supervision was promoted.^14^ This ‘community care’ was often called ‘community control’, its nature conditioned by the original segregationist designation of ‘mental deficiency’ as the antithesis of citizenship.^15^ Indeed, the families of ‘mental defectives’ remained suspect in terms of heredity, as well as practical and moral training.^16^

The inter-war mental deficiency system represents an unprecedented coercive and interventionist strategy for ‘public welfare’.^17^ It was premised on mentally deficient people’s supposed threat to the community, and their inability to perform the role of self-sustaining, responsible citizens.

### 1.1 The Post-war Mental Hygiene Movement

Post war, the conceptualisation of mental deficiency as the antithesis of citizenship began to unravel. However, many of the assumptions associated with it continued to inform both the mental hygiene movement and the mental deficiency system.

In 1946, the NCMH, the CGC and the CAMW merged to form the NAMH, the leading post-war voluntary organisation working for mental hygiene. Its Council comprised mostly doctors and other professionals representing professional organisations. Based at Queen Anne Street near Harley Street, London, the NAMH’s staff included psychiatric social workers and educational psychologists as well as lay workers. Initially led by a psychiatric Medical Director, from the early 1950s the staff operated under General Secretary Mary Appleby with the guidance of an honorary medical panel.

This amalgamation took place alongside the newly elected Labour Government’s institution of a Welfare State that was intended as a final break with the Poor Law. Social protection was to be a right of citizenship with universal and freely accessed services. But, as Mathew Thomson has noted, ‘the impact of ‘democratization’, ‘universalism’, and ‘social citizenship’ was mediated by status’. The NHS shifted power to central bureaucrats and the medical profession.^18^

The principal role of doctors and medicine was reinforced under the NHS Act. Institutions became hospitals. Their administration became separated from community care provision. Local authorities were given permissive powers to provide services in the community. But, as they had formerly operated the institutions, community provision was minimal, and with powers only permissive, little was achieved.

Meanwhile, largely in response to the war, the mental hygiene movement’s concern about the social ‘threat’ of mental deficiency receded. Instead, it increased its focus on emotional maladjustments in the wider population. Nevertheless, the movement did not fundamentally question the role of the mental deficiency system. People deemed mentally deficient continued to be considered incapable of the social responsibility and citizenship that mental hygienists associated with mental health. Throughout the 1940s and 1950s, the mental deficiency system remained coercive and custodial.

Criticism came from elsewhere, however. The National Council for Civil Liberties’ (NCCL) lengthy campaign against the workings of the Mental Deficiency Acts is well known.^19^ From the late 1940s and through the 1950s, it criticised the 1913 Act’s widening of ‘mental deficiency’ to include people termed ‘feebleminded’. Highlighting the inherent class and moral biases in this, the NCCL argued that many of these people had illegitimately lost their rights and citizenship. But the campaign’s impact was wider than this. Its evidence of abuses and failings within the system of certification and licensing, along with the poverty of rehabilitative measures, made public the authoritarian and punitive nature of the system in general.

Although the NAMH was aware of the long waiting-lists, lack of beds, and general poverty of conditions and staffing, it publicly refuted the NCCL’s claims. It maintained that the NCCL was prejudiced, and its focus on ‘wrongful detention’, outdated since the modern mental deficiency institution, was not based on ‘permanent detention’ but on training. However, reaction behind the scenes was more complicated. Conflicting views found common ground in the need to free up places in overcrowded institutions. The NAMH decided that greater hostel provision and more generous licensing might achieve this and began to look at possibilities for amending the legislation in general.^20^

But a number of other factors were also clearly prompting the NAMH to alter its position. One was the growing influence of psychologists. Although hardly any were directly employed in mental deficiency hospitals, from the early 1950s a number began experimental research within them. This played a significant role in psychologists’ assertion of a professional role during the 1950s and 1960s.^21^ It was also made use of by the NCCL. Results demonstrated that the great majority of people in institutions were no danger to society. They also revealed that IQs were often higher than supposed, and that people categorised ‘feebleminded’, as well as those considered ‘imbecile’, had a higher capacity to learn and work than the results of the current system suggested. The NAMH, in fact, helped publicise this research, inviting speakers to its Annual Conferences and publishing articles in its journal *Mental Health*. Indeed, the psychologists involved in this research increasingly worked with the NAMH, promoting new forms of care and rehabilitation. The NAMH was therefore more amenable to reforming the mental deficiency system than its public dismissals of the NCCL’s allegations implied.

Another factor was that the mental hygiene movement had itself developed a critique of institutionalisation. This was derived from the movement’s psychotherapeutic focus on mental health in terms of emotional adjustment and maladjustment. Increasingly, enforced living in large groups within institutions, as well as rigid authority and hierarchy, was considered to be detrimental to human relationships, and therefore to the emotional adjustment of inmates. Instead, the movement began to encourage more open communication and greater freedom of choice within a more egalitarian structure. These principles informed the mental hygiene movement’s promotion of ‘social therapies’ and ‘therapeutic community’ ideas intended to modernise mental hospitals. They also informed the movement’s strong influence over the 1945 Curtis Report on the Care of Children Deprived of a Normal Home. This had set the foundations of post-war policy for the residential care of children. It considered many existing institutions authoritarian, and therefore insensitive to children’s emotional needs, while life in large groups, in particular, created emotional and behavioural problems. But the psychotherapeutic ideas that underpinned these principles had a strong developmental component that explicitly discriminated against most people considered to be mentally deficient. Therapeutic community approaches were not thought to be appropriate for mentally deficient people, and the Curtis Committee considered mentally deficient children to be outside its remit.

Nevertheless, in the context of the NCCL campaign, the prominence of such principles reinforced the view that the mental deficiency system was out of date and custodial.^22^ More significantly, the psychologists researching in mental deficiency, such as H.C. Gunzburg, A.D.B. Clarke and Jack Tizard, applied mental hygienist’s concerns regarding emotional development and adjustment to people detained as feebleminded.^23^ Accordingly, psychologists began to consider institutionalisation for these people, an element of emotional deprivation, involving ‘loss of initiative and personal identity’.^24^ Notably, the NAMH published this research in its own journal, *Mental Health*.

Alongside these developments was the growing influence of the National Association of Parents of Backward Children (NAPBC, now Mencap) which had been founded in 1946. Its affiliated local associations were slowly bringing pressure for services to be more responsive to parental needs.^25^ The NAMH developed a relationship with the NAPBC during the 1940s and 1950s.

The impact of all of these factors on the relationship between citizenship rights and a diagnosis of mental deficiency was ambiguous. While the NAPBC pressed for wider service provision and, increasingly, sponsored research experiments, it was effectively the views of parents rather than mentally defective people themselves that were becoming more prominent. Meanwhile, under a post-war settlement that relied upon ‘full employment’, psychologists’ research on IQ, learning capacity and ability to perform industrial tasks implied the potential reinstatement of citizenship for many people detained in institutions. However, it also suggested that citizenship rights were dependent upon an ability to perform often repetitive and poorly remunerated work.^26^ Indeed, there remained a sense that citizenship was dependent on the capacity of individuals to be improved in order to work and assimilate with the wider population. Similarly, ideas on emotional wellbeing and development were largely limited to ‘high-grade’ patients and translated into a need for training in ‘socialisation’.

Nevertheless, other emphases were also present. Tim Stainton has pointed out that the idea of social rights under the Welfare State certainly contributed to the influence of the NCCL’s campaign.^27^ The psychologist Jack Tizard argued in 1954, at the Royal Medico-Psychological Society AGM, that the concept of ‘high-grade mental defect’ should be abandoned. He argued that these people would be better accommodated under the general post-war welfare services legislation. Indeed, he went further, arguing that the ‘Education Act should be extended to cover “imbecile” children…thus imposing on the educational authorities the responsibility of providing educational training for all children who can benefit from it’. The ‘concept of normality should be broadened’, he said. Two years later, the NAMH published the talk in its journal *Mental Health*.^28^

The 1957 Report of the Royal Commission on the Law Relating to Mental Illness and Mental Deficiency expressed much of this ambiguity regarding mental deficiency and its relationship with citizenship. Although its appointment in 1954 had been spurred by the NCCL’s campaign, it was also considered an overdue attempt to realign the mental illness and deficiency legislation with the post-war Labour Government’s introduction of comprehensive and freely accessed Welfare State services.^29^

The Commission’s terms of reference were to make recommendations on the possibility of treating patients informally without certification. This was a principle around which the NCCL and the NAMH could, in fact, find common ground. While it was critical of the diminished legal safeguards for admission, the NCCL nevertheless welcomed the emphasis on voluntary admission with its reduction of the penal image of care and treatment. The NAMH also welcomed this emphasis. In fact, its precursor organisations, along with the wider mental hygiene movement, had advocated this before the war. Yet this had been on the grounds that the necessary institutionalisation of mental defectives was hampered by certification procedures that pandered to concerns about the liberty of the subject. Nevertheless, the post-war Welfare State emphasis on universal, freely accessed health and welfare services provided a new context for the NAMH’s promotion of voluntary admission. Similarly, it was within this context that both the NAMH and the NCCL were able to support the Commission’s assumption that psychiatry should be assimilated with general medicine.

This meant, however, that mental deficiency (re-labelled mental subnormality by the Commission) was subsumed under the term ‘mental disorder’, and remained within the conceptual and practical setting of ‘illness’ and ‘treatment’. Nevertheless, the Commission accepted that there were debilitating effects caused by institutionalisation. It maintained that this should be avoided by making community care the preferred option with hospitals taking patients only when necessary in the interests of treatment.^30^ Indeed, in accordance with the general thrust of post-war Welfare State services that aimed to maintain citizenship by their universality, non-stigmatising and non-segregationist nature, the Commission’s proposals were formulated into a policy that envisioned a major shift towards community integration of large numbers of patients.

The ambiguities regarding the citizenship status of mentally subnormal people inherent within the Royal Commission’s proposals were enshrined within the 1959 Mental Health Act. Consequently, they continued to be expressed through the 1960s.

The NAMH welcomed the Royal Commission’s Report but was concerned that the proposed wide extension of community care services should have adequate funding, trained staff and an efficiently coordinated overall system. It was disappointed, in particular, that the ensuing 1959 Mental Health Act did not make the development of community care services mandatory on local authorities as the Commission had proposed. The NAMH pressed for investment in community care services and staff training throughout the 1960s.^31^ Yet, in terms of the relationship between mental subnormality and citizenship, the NAMH’s views were equivocal. For instance, psychologists became more prominent within the NAMH during the 1960s. The informational booklets they produced emphasised that the needs of mentally subnormal people were social, educational and occupational, rather than primarily medical.^32^ However, those on ‘social training’ also emphasised training people so that they could assimilate as inconspicuously as possible.^33^.

Citizenship rights were, however, implicit in one important research experiment funded, not by the NAMH, but by the National Society for Mentally Handicapped Children (NSMHC, formerly NAPBC). The influence of the Brooklands experiment on child (and adult) care is commonly cited. Yet, commentators have not drawn attention to the equally important assertion of citizenship inherent in its research design. Headed by Jack Tizard, Brooklands was a home set up for ‘imbecile’ children then living in the Fountain mental deficiency hospital. It explicitly applied the principles that had informed the 1946 Curtis Committee on the Care of Children without a Home. Close, affectionate care was pursued and continuity of relations between particular staff and children attempted. Emphasis was placed on the children’s existing emotional needs, rather than the then prevailing attention on education and training.^34^

This effectively detached the traditional mental hygienist linkage of the means of care and treatment from the aim of producing socially ‘responsible’ and economically productive citizens under the banner of ‘mental health’. These ‘imbecile’ children were unable to attain this goal, but the inherent assumption of the research design was that this should not be allowed to deny their right to the same quality of care and support as other children. More fundamentally, Brooklands foregrounded similarities of emotional experience and response instead of difference and deficiency. This in itself was a powerful statement of shared humanity and citizenship.

It bears pointing out that the staffing and material provisions made available for the experiment itself were inadequate, especially in its first year. However, conditions at the hospital from which the children had come were themselves poor, with harassed nurses working on wards of around sixty beds. Indeed, these conditions were commonplace at mental deficiency hospitals at the time. Potentially more disquieting is the lack of information about the fate of the children after the experiment. It appears unlikely that there was a continuation of the care that it was asserted they were entitled to.

Notwithstanding these reservations about the experiment in practice, Brooklands inherently asserted citizenship rights. And it did so, on the basis of mental hygienist psychotherapeutic theories that had originally excluded people considered mentally deficient.

In the 1960s, Tizard and colleagues, Norma Raynes and Roy King, extended the Brooklands research, highlighting the poverty of ‘institutionally oriented care’ in contrast with ‘inmate oriented’ care based on the Curtis-style ‘family’ model.^35^ The 1967 Wessex Project, set up by Tizard with the psychiatrist Albert Kushlick, employed the same differentiation to develop a series of small community residential homes for children and adults categorised as severely mentally handicapped.^36^

The psychiatric profession has been represented as resisting criticism of mental subnormality hospitals. But there were mixed views among those associated with the NAMH. Both Alexander Shapiro, a member of the NAMH’s Mental Deficiency Sub-Committee from the mid-1950s until the early 1960s, and Alan Heaton-Ward, a medical adviser to the NAMH from 1967, repudiated what they considered unjust attacks on hospitals and psychiatric leadership over care and treatment. Other psychiatrists, however, considered hospitals to be still custodial, hierarchical and institutionalising, arguing that around half of residents did not need nursing or medical facilities.^37^

By 1966, the NAMH had embraced this latter view, noting that it had ‘been estimated half…could leave hospital if suitable accommodation and support was available in the community’. This signalled a more forthright stance. In the same year, it confessed, ‘We have, in our anxiety not to do harm, remained silent or at least discrete, about conditions which we knew to be bad.’ Along with underfunding, long waiting-lists and inappropriate buildings, the NAMH admitted that ‘long-stay patients are left virtually untreated’.^38^ The NAMH began to criticise the management of some hospitals. In 1967, it called for more research on the workings of the Mental Health Review Tribunals (MHRTs), and began working with NCCL to design a patient representation service.^39^

## 2 The Emergence of MIND and its Rights-based Critique

The NAMH’s admission was prescient. A series of hospital scandals broke out in 1967. Alongside the developing critiques of hospital-based services, these pushed the NAMH into an even more critical and assertive position. *Sans Everything*, a book with a foreword by the psychiatrist Russell Barton, a prominent member of the NAMH, alleged serious abuse and neglect on wards for elderly people in several hospitals.^40^ A scandal emerged at Ely Hospital in Cardiff involving abuse, along with generally poor treatment and conditions. Further concerns about conditions and treatment in hospitals followed the next year. In 1969, Richard Crossman the Secretary of State for Health and Social Services, had the official report on Ely published in full, despite resistance from his own Ministry.^41^ That same year, the sociologist Pauline Morris published a large-scale survey of thirty-five mental subnormality hospitals funded by the NSMHC from 1964. This described severe overcrowding in often dilapidated buildings, with very poor staff–patient ratios, poor training and inadequate staff communication.^42^ NSMHC’s sponsorship reflected its increasing concern about hospital conditions. It was a founder member of the International League of Societies for the Mentally Handicapped which, in 1968, issued a Declaration of General and Special Rights of the Mentally Retarded.^43^

The NAMH’s immediate response to the scandals was to develop a scheme for an independent inspectorate of hospitals to present to the Minister.^44^ Its more general response was to embark on a ‘consumerist’ and rights-based campaigning role. This would ultimately transform its original mental hygienist equation of mental deficiency with the antithesis of citizenship into an approach that placed the citizenship of people with learning disabilities at the centre of community-based health and welfare services.

In 1970, the NAMH announced its intention to begin a major national campaign.^45^ It aimed, in particular, to highlight the problems of institutional care and of insufficient provision within the community. Regarding mentally handicapped people (as they were now commonly being termed) the NAMH maintained that:

Our main concern is with the individual and his mental health, whatever his innate intellectual capacity. That the ability of the mentally handicapped to enjoy life should not be impaired by a lack of human warmth, appropriate assessment and every opportunity for self-fulfilment.^46^

This statement marks a milestone in the NAMH and the mental hygiene movement’s policy. It is a tacit recognition that intellectual capacity cannot, in itself, be a measure of mental health. Simultaneously, it represents an acceptance that the mental hygienist equation of mental health with adequate citizenship and ‘social efficiency’ had become subverted.

The NAMH admitted that there were fundamental questions regarding hospital services:

The concept of treatment for the mentally handicapped in hospitals embodied in the Mental Health Act has never been sufficiently discussed. What is this treatment? Who should undertake it? To what extent are these hospitals ‘homes’, and if homes, how ‘homelike’ are they?^47^

Regarding severely handicapped children living in hospital it asked, ‘Have the findings about maternal deprivation, so familiar to the child-care world, been applied to the grossly handicapped child in hospital?’^48^ Fundamentally, these concerns mark a tacit (if belated) acceptance that the theories of emotional development and emotional wellbeing, so influentially promoted by the mental hygiene movement, should not exclude mentally handicapped children or adults, whatever their ‘grade’.

NAMH’s 1971 ‘MIND Campaign’ self-consciously employed the language of rights. Although unmentioned by the NAMH, the 1968 Declaration of General and Special Rights of the Mentally Retarded clearly provided a backdrop to this shift. A more direct influence on the NAMH was an ongoing conflict with the Church of Scientology that had begun earlier in the 1960s. The Scientologists used human rights terminology to aggressively attack the psychiatric notion of mental health and disorder, increasingly targeting the NAMH itself.^49^ In contrast, the NAMH’s ‘MIND Manifesto’ declared that ‘Every citizen’ had a ‘right to demand that mental health be given as high a priority as physical health’. All whose job it was to care for the mentally disordered had a right to adequate working conditions, and families had the right to care for a ‘disabled member’ at home with support or know that an acceptable alternative was available.^50^

In fact, the terminology of rights had become prominent within broader debates about the organisation of welfare in conjunction with the notion that this was the era of social rights. Popularised by T.H. Marshall in his 1950 *Citizenship and Social Class*, social rights were considered a collectivist matter, intrinsic to the post-war Welfare State.^51^ By the 1960s, with deficiencies in health and welfare services increasingly apparent, attention began to focus on legal aid provision. As Tamara Goriely has emphasised, legal aid schemes have a dual role: they are an aspect of welfare provision, but they are also a means to enforce welfare rights.^52^ The legal Aid and Advice Act had been passed in 1949. However, during the 1960s there was growing pressure to extend the limited remit of legal aid for courts and to tribunals.^53^ By the early 1970s, activists were attempting to give legal aid a principal role in gaining access to justice for poor and marginalised people.^54^ Legal and welfare rights were asserted by the emergence of self-help and pressure groups such as Tenants’ Associations and Claimants’ Unions.^55^ The Child Poverty Action Group (founded in 1965 in response to the Labour Government’s failure to increase family allowances) opened a legal department in 1969 and, in 1970, a Citizen’s Rights’ Office. During the 1970s, it turned to litigation as a means to test the interpretation of social welfare law.^56^ Under its Director Tony Smythe, the NCCL promoted legal aid and advice for marginalised and disadvantaged groups in the later 1960s.^57^ As noted, in 1967 the NAMH and the NCCL had attempted to combine their expertise in the creation of a MHRT representation scheme.

It is within this complex that the emergence and mobilisation of the NAMH’s rights-based thinking needs to be situated. For example, in 1972 it collaborated with the Disablement Income Group (DIG) and the Spastics Society to press for patients to receive pocket money as of right under social security legislation instead of at the discretion of hospital authorities.^58^ But the NAMH struggled to appreciate the underlying issues regarding citizenship and associated questioning of the social organisation of health and welfare services. This conflict can be illuminated by examining the NAMH’s position in contrast with the Campaign for the Mentally Handicapped (CMH). The CMH emerged from the *Guardian* welfare journalist Ann Shearer’s experience of visiting mental handicap hospitals. Her response was to prepare a manifesto calling for their closure. This drew hostility from many psychiatrists. But, with the support of Anita Hunt, a researcher at the Spastics Society, and Sandra Franklin, an architect, the CMH was launched in early 1971.^59^ In the same year, the CMH sent a document of its aims (probably Shearer’s ‘manifesto’) to the NAMH asking for its support. The NAMH felt unable to approve the central proposition that hospitals should be closed, but sympathised with other aspects of the document.^60^ In fact, it was willing to distribute, and reprint in its magazine, a pamphlet called ‘A Right to Love?’ by Shearer which criticised institutional living and segregation.^61^

But the gulf between the CMH and the NAMH can be appreciated in the contrasting views on what to do about services for mentally handicapped people published in the first issue of *Apex*, the journal of the newly founded Institute of Mental Subnormality.

Mary Appleby, the NAMH’s General Secretary, forcefully attacked the polarisation of views between the ‘hospital lobby and the lobby in favour of community care’. She argued that there should be ‘a single focus of leadership’ which would transcend ‘artificial distinctions between hospital and community, between treatment and care’. But this was to be done by enabling hospitals to regain the leadership role that they had lost. This ‘power unit’ would be the ‘centre of a series of concentric circles of complex supportive provision’. Here the psychiatrist would be able to call on the skills of other medical, psychological, educational and social workers. It would ‘concentrate the sum of technological knowledge on the most difficult patients’, guide medical treatment, devise training programmes and educate parents and staff. Appleby argued that this should be a completely separate service for mental handicap, able to command financial support from government through a single channel.^62^

Ann Shearer’s combative article offered a very different vision. She maintained that people’s attitudes to mental handicap mirrored their deeper attitudes to society. Confronting them would

…force us to ask whether this is to be a society which accepts people of different abilities and the economic dependence which goes with this, or one which shuts them away from its normal patterns in however kindly intentioned a seclusion.…To recognise their rights as full citizens and to work towards realising these rights – even though they have never, unlike the elderly been economically productive, and they may never, unlike the disadvantaged child, repay our investment in them by becoming economically productive – is a very deep challenge to a materialist society.

Shearer, emphasised that there had been no fundamental questioning of the role of hospitals in providing care that was now recognised to be social and educational and only primarily medical for a small number of people. Meanwhile, vague formulations of ‘community care’ had left families providing the essential and unpaid services that they always had.^63^

The CMH promoted an egalitarian ethos, aiming to involve and work alongside learning disabled people, with Shearer advocating therapeutic community-style approaches.^64^ As Peter Barham has noted, the egalitarian ideals of ‘social therapy’ resurfaced and were refashioned in challenges to welfare paternalism and the lack of voice of service users.^65^ This is important. The mental hygiene movement had been prominent in the development of social therapies, therapeutic communities and an associated critique of institutionalisation as inhibitive of wellbeing. But the psychotherapeutic theories underlying these discriminated against application to mentally deficient people. Only gradually, and predominantly through the work of psychologists, had the movement accepted their relevance. In any case, the movement had considered therapeutic community and social therapy approaches as modernising treatment technologies, not fundamental challenges to hospital-centred care. Nor did the movement aim to develop any potential implications for revising notions of citizenship and its relationship to health and welfare.

Such a revision was exactly what Shearer and the CMH were pointing to. And it was this that the mental hygiene movement could not adapt itself to. The period from the end of the 1960s, in fact, marked the collapse of the mental hygiene movement. Its conceptualisation of mental illness and mental deficiency (by the 1970s, usually referred to as mental handicap) in negative relation to ‘social efficiency’ and ‘responsible’ citizenship had become undermined.

In 1972, the NAMH decided to adopt the ‘brand-name’ MIND and permanently retain its rights-based, pressure group campaigning.^66^ Faced with this, the organisation’s medical advisers stepped down from their roles but offered to provide advice when called upon.^67^ The transition ultimately alienated many psychiatrists and a number of other supporters. By January 1974, MIND had appointed Tony Smythe from the NCCL as Director. It now pursued a more forthright rights-based agenda. In 1975, a Legal and Welfare Rights Department was created, headed by a young American civil rights lawyer, Larry Gostin. This is the period on which commentators on MIND’s rights campaigning have focused. In general, MIND has been represented as fundamentally opposed to medicine and psychiatry, framing debate in terms of an individualist concern with the deprivation of liberty.^68^

Yet, our understanding of MIND’s approach is inhibited by reducing it to individualism in antagonism to medicine and psychiatry. The Smythe era at MIND is better characterised as one in which the aim of rights-based campaigning was more finely focused in an attempt to reconstruct the citizenship rights of mentally handicapped people around reconfigured and comprehensive health and welfare services.

One reason why contemporary critics of MIND’s legal-rights campaigning focused only upon the mental illness aspects of its work may be that they concentrated on the analysis and proposals for reform of the 1959 Mental Health Act set out in Volume one of *A Human Condition*, published in 1975.^69^ Although the provisions of the Act dealt with both mental illness and ‘mental subnormality’ under the overall term ‘mental disorder’, *A Human Condition* was mainly oriented towards provisions for people diagnosed mentally ill. Its contention that the voluntary basis of admission could be rendered nugatory by the continued presence of coercive powers, patient passivity or even ‘induced collusion’ was heavily criticised.^70^ Yet this ignores the large majority of the 60 000 people living in mental handicap hospitals who were ‘voluntary’ patients. As Gostin observed in a footnote, ‘The issue of informal admissions for mentally handicapped people is especially well-defined. It is not often that these persons will actively object to their confinement, even though the confinement is not necessarily in their own best interests.’^71^

The health and welfare of mentally handicapped people was an important part of MIND’s rights-based campaigning. Indeed, even while *A Human Condition* was being published, MIND was working closely with the CMH and beginning to elaborate a framework for community-based services. Over the next few years, in evidence and responses to various government committees, MIND outlined the coordinates of a comprehensive health and welfare scheme that had social integration and citizenship rights at its heart.^72^ The transformed approach was expressed by the opening line of MIND’s evidence to the Jay Committee into Mental Handicap Nursing and Care, which had been set up in 1975: ‘Over the years, the constant cry from those campaigning in the field of mental handicap has been “more”. While more (finance and manpower) is certainly needed we would add “yes, not only more, but different”.’^73^

In 1976, the government set up a Royal Commission on the National Health Service to look into the financing and personnel resources of the NHS. MIND’s evidence to the Commission represents the summation of MIND’s rights-based approach to mental handicap.^74^ The working party formed to compile this evidence included Smythe and other MIND staff, Maureen Oswin of the CMH, a parent, two consultant psychiatrists, teachers and a lecturer in social work. The psychologist A.D.B. Clarke, who had carried out ground-breaking research work since the 1950s and had written the NAMH’s booklet *Recent Advances in the Study of Mental Deficiency*, was among others, including two consultant psychiatrists, who provided advice and additional material.

MIND’s evidence quoted from its former General Secretary Mary Appleby’s article on the mental handicap hospital as a ‘power house’, citing her comment that, despite optimism over the 1959 Act’s liberal ethos, few had foreseen ‘what damage would be done to planning for mental handicap by forcing it into the straightjacket which really applied to the psychiatrically ill’. But MIND now used this to emphasise the erroneousness of the implication that mental handicap was a ‘disorder’ or sickness capable of ‘treatment’ by medical and nursing staff. Mentally handicapped people had a ‘life-long disability’ and should be helped and supported through ‘education, training and socialisation’.^75^ MIND advocated the kind of approaches to residential provision proposed by Jack Tizard at Brooklands, along with his colleagues Norma Raynes and Roy King, and put into practice with Albert Kushlick’s work in Wessex.^76^ This showed, MIND emphasised, that care and integration within the community could be provided for all including the most severely mentally handicapped people.^77^

MIND argued that mentally handicapped people should be removed from the scope of the 1959 Mental Health Act, unless they also had a mental illness, and argued that existing legislation could be used, or amended, in order to achieve this goal. In its ‘Further Evidence to the Royal Commission on the National Health Service’ in 1979, MIND concluded that:

…*there is an urgent requirement for an enabling Act which would set out the right of mentally handicapped people to education, training, employment, accommodation, support, rehabilitation, social care and, where necessary, protection.* These services would then be provided under existing legislation within which, education, welfare, employment, income-support and health services are provided to the population as a whole.^78^

This integrationist stance represents, in effect, an extension to all mentally handicapped people, of the argument made by Tizard back in 1954 regarding people labelled ‘feebleminded’ (and partially those termed ‘imbecile’). MIND maintained that mentally handicapped people and their families had the same needs as other citizens, as well as some special requirements. No single service or administrative structure could cater for all these. It was illogical to expect hospitals to ‘respond to the whole range of [their] human needs’.^79^ Comprehensive services should be built around, and made responsive to, the assessed needs of mentally handicapped people and their families. These would provide ‘financial help, accommodation, education, domiciliary services, advice and practical help from social workers, community nurses and other therapists, regular respite from the burden of care and, in cases where families can no longer cope, as normal an alternative to family care as is feasible’.^80^ MIND remarked that, ‘Integration and normalisation are principles which demand choice, and unfettered access to services used by all other citizens.’ It added that, ‘It is because the “general need” services have failed mentally handicapped people so shamefully that segregated and inferior services have continued so long in existence.’^81^

This vision required a complete restructuring of the service and brought MIND into conflict with the medical profession. MIND sought to close all the large, long-stay hospitals, with the released resources used to develop community-based services. These would be mainly part of the local authority social and family support services but include NHS services where appropriate. The role of the consultant psychiatrist would be redefined ‘to bring to the community the knowledge and skills of a doctor and a psychiatrist as they relate to mental handicap’. This would represent a reduced role in terms of authority and remit. Psychiatrists would focus on assessment, visiting and treating within families or community homes, and preventing unnecessary hospitalisation. MIND argued that a true interdisciplinary approach was impossible in the mental handicap hospitals because, although all the services may be on one site, the patient had little control over what happened. Overall responsibility rested with the consultant and senior nursing staff.^82^ MIND wanted greatly increased roles for professionals in education and psychology, and their membership, along with parents, within a ‘democratic’ multi-disciplinary team. It argued that nurses were already playing an important role in some community-oriented services, but that along with transformed training, attitudinal change was especially important.

But MIND’s proposed community service would involve extending the work of other medical professionals so that mentally handicapped people and their families would have the same access to general medical services as other citizens. MIND stressed the need for increased training and input from community paediatricians and general practitioners. It argued that medical services should concentrate on primary care, and on ‘the important task of applying research screening techniques and preventive measures to reduce the incidence of mental handicap in its most severe forms’. Indeed, MIND maintained that the lack of attention on these areas by the medical profession illustrated the failure of the prevailing system.^83^

At the root of these comprehensive community-based proposals were twin themes. One was the goal of integrating mentally handicapped people and their families ‘into the daily life of ordinary people’ and providing them with as much control as possible over their own services. The other was that the ‘relative powerlessness and inarticulateness of mentally handicapped people‘ meant that their legal and welfare rights were ‘easily disregarded’. MIND maintained that it was essential for mentally handicapped people and their families to be ‘enabled to participate in the planning and running of services and to take a substantial part in arriving at major decisions’.^84^

## 3 Conclusion

Analysis of MIND’s historical origins within the mental hygiene movement illuminates the fundamental reconceptualisation of citizenship and learning disability that took place in the twentieth century. Turn of the century anxieties, among some, about ‘adjusting to democracy’ had focused concern on mental deficiency. Along with associated eugenic concerns, this cast these people as the antithesis of citizenship, due to innate deficiency and social inefficiency. Institutionalisation and social control became the watchwords. The inter-war mental hygiene movement maintained this view, while nevertheless increasingly placing its greatest concern on the mental stability of the wider population.

Overstated, the early post-war decades might be summed up in these terms. After the war, mentally deficient people ceased to be considered a threat to society, but few talked about it. In the process, they became citizens, but few noticed. The mental hygiene movement shifted away from vocal concern about the social threat of mental deficiency in the immediate post-war period, but it did not reconsider the fundamental nature and aims of the mental deficiency system. Meanwhile, the inauguration of the Welfare State did little to improve the citizenship status of mentally deficient people, building on the services that had existed before the war and drawing these into the NHS. Additionally, the experiments by psychologists during the 1950s, much cited in the historical literature as significant for changing perceptions towards mentally deficient people, were, in fact, ambiguous regarding citizenship. While confirming their lack of threat to society, the bulk of the research revealed unrecognised abilities to learn and perform routine tasks. This suggested that many people could attain some place in society. But it nevertheless implied that citizenship relied on ‘improving’ these people so that they could assimilate into the ‘lowest’ work levels.^85^ This left the nature of citizenship rights unexamined. Indeed, the general failure to directly address the relationship of citizenship to mental deficiency helps account for the ambiguities of the 1959 Mental Health Act and subsequent 1960s developments.

Conventional histories of post-war learning disability make little reference to psychotherapeutic models; understandably so. Their direct impact has appeared insignificant and more general impact negative. Yet, despite the conceptual bias against people considered mentally deficient, the present analysis shows that, from the 1950s, a discourse of emotionality derived from psychodynamic thinking did gradually have a positive impact. Under the influence of psychodynamic ideas, mental hygienists developed a general concern with emotional development and wellbeing, emphasising emotionality as a dynamic relational phenomenon, deteriorated by insensitive relationships and rigid institutional life. This occurred largely through application by professionalising psychologists, at a time of sustained criticism of the mental deficiency system by the NCCL. Despite the potential ethical issues of the experiment itself, the later Brooklands research expressed a powerful statement of shared humanity and citizenship. Instead of difference and deficiency, it highlighted similarities of emotional experience and response. These children were less a separate class than ‘one of us’. Their rights of citizenship should therefore not depend upon an ability to become socially ‘responsible’ and economically productive citizens. MIND and the CMH drew directly on this and the subsequent 1960s work with adults.

MIND aimed to place the citizenship rights of people with learning disabilities at the heart of comprehensive community-based health and welfare services. The NAMH had been unable to make this shift within the discourse of mental hygiene and the accompanying need to accommodate the professional interests represented within it. But, clearly, MIND’s campaigning cannot be reduced to an individualist approach that pitted itself against medicine and psychiatry and founded its stance on considerations of the deprivation of liberty. Some in the psychiatric profession, nevertheless, considered it so. For instance, in 1984, Kenneth Rawnsley, in a Presidential address to the Royal College of Psychiatrists, maintained that the attempt to remove mental handicap from the remit of the Mental Health Act (which he reduced to a campaign by Mencap) ‘boiled down to a reluctance to allow mental handicap to appear in an Act of Parliament, alongside mental illness, since this would somehow stigmatise the former condition’.^86^ But this is a caricature. MIND, the CMH and Mencap all sought mental handicap’s removal on the basis of research and practical evidence that had accrued since the 1950s. Heaton-Ward, a former medical adviser to the NAMH, in similar vein, accused the CMH of promoting the idea that mentally handicapped people should be treated ‘as though they were, in fact, normal’.^87^ This is untrue. Along with MIND, the CMH provided detailed analyses of the types of support and care necessary for a community-based service, and set out sophisticated analyses of the internal social organisation necessary within residential provision and the make-up of multi-professional services.

Neither can MIND be justifiably considered opposed to psychiatry and medicine. Given that it severely criticised the prevailing medically oriented services for mentally handicapped people and sought a reduced role and authority for psychiatrists within the community, it is understandable that many doctors took this line. But MIND wanted what it considered a more cogent role for psychiatrists within a community service based on citizenship rights. And, indeed, it sought to extend the role of general practitioners and paediatricians. Yet, MIND could be criticised for underestimating the extensive difficulties – political, professional, economic, organisational and attitudinal – of so comprehensively reorienting the support, care and treatment of mentally handicapped people. Taking place over a period of economic and political turmoil, MIND’s campaign had only limited success. The Royal Commission on the NHS paid little regard to its evidence regarding mentally handicapped people. But MIND had greater success with the reform of the 1983 Mental Health Act. Mentally handicapped people were effectively removed from its remit unless they also suffered from a mental health problem. The 1979 Jay Report on Mental Handicap Nursing and Care can also be considered a success for MIND in that its radical proposals for reoriented care were founded on community-based citizenship rights, allowing people to lead as normal lives as possible and interpreting primary needs to be social and educational. Yet, as MIND predicted, the impact of this report on services was long delayed by the government-imposed expenditure cuts in the 1980s.^88^ Indeed, it was not until 2001 that a government policy paper forthrightly expressed a strategy based on inclusive citizenship.^89^ The extent to which the lives of people with learning disabilities and their families have been affected by this transformation in policy remains debatable. Nevertheless, while the CMH has justifiably been acknowledged as an early and influential promoter of citizenship rights and advocacy, MIND too deserves recognition.^90^

## Footnotes

1.
For example, John Welshman and Jan Walmsley (eds), *Community Care in Perspective: Care, Control and Citizenship* (Basingstoke: Palgrave MacMillan, 2006); David Race, ‘The historical context’, in David Race (ed.), *Learning Disability; A Social Approach* (London: Routledge, 2002).

2.
Nikolas Rose, ‘Unreasonable Rights: Mental Illness and the Limits of the Law’, *Journal of Law and Society*, 12, 2 (1985), 199–218; Nikolas Rose, ‘Law, rights and psychiatry’, in Nikolas Rose and Peter Miller, *The Power of Psychiatry* (Cambridge: Polity Press, 1986), 177–213; Kathleen Jones, *Asylums and After: A Revised History of the Mental Health Services: From the Early 18th Century to the 1990s* (London: The Athlone Press, 1993).

3.

Peter Sedgwick , *Psychopolitics*, ch. 7 (London: Pluto Press, 1982); Rose, *op. cit.* (note 2).

4.
For an overview, see Katja Guenther and Volker Hess (editorial), ‘Soul Catchers: The Material Culture of the Mind Sciences’, *Medical History*, 60, 3 (2016), 301–7.10.1017/mdh.2016.24PMC490432227292321

5.
Alexandra Bacopoulos-Viau and Aude Fauvel (editorial), ‘The Patient’s Turn: Roy Porter and Psychiatry’s Tales, Thirty Years On’, *Medical History*60, 1 (2016), 1–18; Sarah Chaney, “‘No ‘Sane’ Person Would Have Any Idea”: Patients’ Involvement in Late Nineteenth-century Psychiatry’, *idem*, 37–53; Hazel Morrison, ‘Constructing Patient Stories: “Dynamic” Case Notes and Clinical Encounters at Glasgow’s Gartnavel Mental Hospital, 1921–32’, *idem*, 67–86.

6.
The mental hygiene movement began in the United States in the first decade of the twentieth century. On this history, see, for example, Johannes Pols, “‘Beyond the Clinical Frontiers”: The American Mental Hygiene Movement, 1910–1945’, in Volker Roelcke, Paul Weindling and Louise Westwood (eds), *International Relations in Psychiatry: Britain, Germany and the United States to World War Two* (Rochester: University of Rochester Press, 2010), 111–33.

7.
It was originally called the Central Association for the Care of Mental Defectives, changing its title to CAMW in 1922.

8.
Mathew Thomson, *The Problem of Mental Deficiency: Eugenics, Democracy, and Social Policy in Britain, c. 1870–1959* (Oxford: Clarendon Press, 1998), 217.

9.
For a recent example, David Stack, ‘A Moment of Madness’, *Total Politics*, 43, 2012.

10.
Thomson, *op. cit.* (note 8).

11.
Thomson, *op. cit.* (note 8), 46–7. That the Act’s extension of ‘mental deficiency’ to include people considered ‘feebleminded’ *did* have a significant class basis was, in fact, emphasised by Josiah Wedgewood, one of the few parliamentarians to strongly resist its implementation.

12.

Greta Jones , Social Hygiene in Twentieth-Century Britain (London: Croom Helm, 1986), 27–28.

13.

John Stewart , Child Guidance in Britain 1918–1955: The Dangerous Age of Childhood (London: Pickering and Chatto, 2013).

14.
See, for instance, Ruth Darwin, ‘The proper care of defectives outside institutions’, in Central Association for Mental Welfare, *Report of a Conference on Mental Welfare Held in the Central Hall, Westminster, London, SW on Thursday and Friday, December the 2nd and 3rd, 1926* (London: CAMW, 1926), 23–35: 23–4.

15.
Evelyn Fox, ‘The Mentally Defective and the Community’, *Studies in Mental Inefficiency*, 4, 4 (1923), 71–9. Wellcome Trust Archives and Manuscripts, Mind Archives: SA/MIN/B/80/57a.PMC510917928910004

16.
Jan Walmsley, Dorothy Atkinson and Sheena Rolph, ‘Community care and mental deficiency 1913–1945’, in Peter Bartlett and David Wright (eds), *Outside the Walls of the Asylum: The History of Care in the Community 1750–2000* (London: The Athlone Press, 1999), 181–203.

17.
Clive Unsworth, *The Politics of Mental Health Legislation* (Oxford: Clarendon Press, 1987), 51–2.

18.
Thomson, *op. cit.* (note 8), 293.

19.
For a fuller discussion of the NCCL’s campaign, the input of psychologists and NAMH’s response, see Jonathan Toms, *Mental Hygiene and Psychiatry in Modern Britain* (London: Palgrave MacMillan, 2013), 120–5: 130–4.

20.

*Ibid*.

21.
John Hall, ‘Mental Deficiency – Changing the Outlook’, *The Psychologist*, 21, 11 (2008), 1006–7; Race, *op. cit.* (note 1).

22.

Mark Lilly , The National Council for Civil Liberties: The First Fifty Years (London: MacMillan, 1984), 78–79.

23.

H.C. Gunzburg , ‘The Colony and the High-Grade Mental Defective’, Mental Health, 9, 4 (1950), 87–92. SA/MIN/B/80/27/10.28908240PMC5078437

24.

A.D.B. Clarke and A.M. Clarke , ‘A Rehabilitation Scheme for Certified Mental Defectives’, Mental Health, 14, 1 (1954), 4–10. SA/MIN/B/80/27/14.28908041PMC5077396

25.

Sheena Rolph , Reclaiming the Past: The Role of Local Mencap Societies in the Development of Community Care in East Anglia (Milton Keynes: Open University, 2002).

26.

Vicky Long , ‘Work is therapy? The function of employment in British psychiatric care after 1959’, in Waltraud Ernst (ed.), Work, Psychiatry and Society, c. 1750–2015 (Manchester: Manchester University Press, 2016), 334–350.

27.

Tim Stainton , ‘Equal citizens? The discourse of liberty and rights in the history of learning disability’, in Lindsay Brigham , Dorothy Atkinson , Mark Jackson , Sheena Rolph and Jan Walmsley (eds), Crossing Boundaries: Change and Continuity in the History of Learning Disability (Kidderminster: BILD, 2000), 87–102: 97.

28.

J. Tizard , ‘The Mental Deficiency Services Today and Tomorrow’, Mental Health, 15, 3 (1956), 85–92: (quote on p. 89). SA/MIN/B/80/27/16.28908069PMC5077500

29.
Unsworth, *op. cit.* (note 17), 255–6.

30.

*Royal Commission on the Law Relating to Mental Illness and Mental Deficiency 1954–1957, Report* (London: HMSO, 1957), Cmnd.169, para. 601.

31.
See, for example, NAMH Annual Report 1963–4. SA/MIN/B/80/7/2.

32.
For example, A.D.B. Clarke, *Recent Advances in the Study of Mental Deficiency* (London: NAMH, 1966). SA/MIN/B/74a.

33.

H.C. Gunzburg , Junior Training Centres: An Outline of the Principles and Practices of Social Education and Training of the Mentally Subnormal Child (London: NAMH, 1963) [reprinted 1966], 10.

34.

Jack Tizard , Community Services for the Mentally Handicapped (London: Oxford University Press, 1964), 101.

35.

Norma V. Raynes and Roy D. King , ‘Residential care for the mentally retarded’ [1967], in D.M. Boswell and J.M. Wingrove (eds), *The Handicapped Person in the Community* (London: Tavistock and Oxford University Press, 1974), 299–306: 299.

36.

Albert Kushlick , ‘The need for residential care’, in V. Shennan (ed.), Subnormality in the 70s: Action for the Retarded (London: NCMHC and WFMH, 1972), 13–26. A.D.B. Clarke and B. Tizard (eds), *Child Development and Social Policy: The Life and Work of Jack Tizard* (Leicester: BPS, 1983), 4.

37.

T.L. Pilkington , ‘Hospital Services for the Mentally Subnormal’, The Lancet, 282 (1963), 1279 (originally published as Vol. 2, issue 7320); T.L. Pilkington, speech, in NAMH, *The Heart of the Matter: Report of the Annual Conference, 1966* (London: NAMH, 1966), 50–7. SA/MIN/B/80/43/4.

38.
NAMH, Annual Report 1965–6, 5; Council of Management Minutes, paper on ‘National Trends and The Mental Health Services’ 21 November 1967. SA/MIN/A/3/1.

39.
NAMH, Annual Report 1967–8, 18. SA/MIN/B/80/7/2.

40.

Barbara Robb (ed.), Sans Everything: A Case to Answer (London: Thomas Nelson & Sons Ltd, 1967).

41.
A White Paper, *Better Services for the Mentally Handicapped*, was published in 1971, under the Conservative Government. Hospital population was to be reduced considerably but hospitals were not intended to be emptied and closed.

42.

Pauline Morris , Put Away: A Sociological Study of Institutions for the Retarded (London: Routledge, 1969).

43.

Brian Rix , All About Us: The Story of People with a Learning Disability and Mencap (London: Mencap, 2006). The Declaration’s seven Articles are reprinted in S. Mattingly (ed.), *Rehabilitation Today* (London: Update Books, 1977), 175. It was adopted virtually unchanged by the United Nations General Assembly in December 1971. However, there remained ambiguity in its provisions regarding the extent of community integration.

44.
NAMH, Annual Report 1968–9, 8; Council of Management, 23 June 1967. SA/MIN/A/3/1.

45.
NAMH, Annual Report 1969–70, 5. SA/MIN/B/80/7/2.

46.

*Ibid.*, 5.

47.

*Ibid.*, 4. SA/MIN/B/80/7/2.

48.

*Ibid.*, 4.

49.

*Ibid.*, 8. SA/MIN/B/80/7/2; C.H. Rolph, *Believe What You Like: What Happened between the Scientologists and the National Association for Mental Health* (London: Deustch, 1973); NAMH, *op. cit.* (note 45). SA/MIN/B/80/7/2; Council of Management Minutes throughout 1967–1972. SA/MIN/A/3/1.

50.
NAMH, *MIND Manifesto*, republished in *MIND and Mental Health*, 1 (1971), 3–6: 4. SA/MIN/B/80/27/28.

51.

T.H. Marshall , Citizenship and Social Class and Other Essays (Cambridge: Cambridge University Press, 1950).

52.

Tamara Goriely , ‘Making the welfare state work: changing conceptions of legal remedies within the British welfare state’, in Francis Regan , Alan Paterson and Tamara Goriely (eds), The Transformation of Legal Aid: Comparative and Historical Studies (Oxford: Oxford University Press, 1999), 89–112.

53.

Anthea Byles and Pauline Morris , Unmet Need: The Case of the Neighbourhood Law Centre (Routledge: London, 1977), 1–4.

54.
Regan *et al.* *op. cit.* (note 52), 1.

55.

Hilary Rose , ‘Up Against the Welfare State: The Claimant Unions’, Socialist Register, 10 (1973), 179–203.

56.
Maria Mayer-Kelly and Michael D. Kandiah (eds), ‘The Poor Get Poorer Under Labour: the Validity and Effects of CPAG’s Campaign in 1970’, ICBH Witness Seminar Programme, seminar held 18 October 2000, (Institute of Contemporary British History, 2003, http://www.icbh.ac.uk/icbh/witness/cpag/, accessed 13 April 2016).

57.

Brian Dyson , Liberty in Britain, 1934–1994: A Diamond Jubilee History of the National Council of Civil Liberties (London: Civil Liberties Trust, 1994), 43.

58.
NAMH, Annual Report, 1971–2, 12. SA/MIN/B/80/7/3.

59.

Paul Williams , ‘The Roots of a True Campaigning Movement’, Community Living, 26, 3 (2013), 17.

60.
NAMH Council of Management, 18 June 1971. SA/MIN/A/3/2.

61.
This was reprinted in NAMH, *MIND and Mental Health*, Summer (1972), 14–17. SA/MIN/B/80/27/29.

62.

M. Appleby , ‘The Hospital as a Power House of Care’, Apex, 1, 1 (1973), 21.

63.

Ann Shearer , ‘Wanted: Political Leadership not ‘Generalised Goodwill’’, Apex, 1, 1 (1973), 20.

64.
Reported in Mary Manning, ‘Do Staff Really Need Qualifications?’, *Community Care*1 October 1975, 7.

65.

Peter Barham , ‘From the asylum to the community: the mental patient in post-war Britain’, in Marijke Gijswijt-Hofstra and Roy Porter (eds), Cultures of Psychiatry and Mental Health Care in Post-War Britain and the Netherlands (Amsterdam: Clio Medica, 1998), 221–240: 229.

66.
NAMH, *op. cit.* (note 58), 7. SA/MIN/B/80/7/3.

67.
Council of Management Minutes, 23 November 1973. SA/MIN/A/3/3.

68.
Jones, *Asylums*, *op. cit.* (note 2), 203; Anthony Clare, ‘Can The Law Reform Psychiatry?’ *MIND OUT*, 48 (1981), 17; Rose, ‘Unreasonable Rights’, *op. cit.* (note 2); Sedgwick, *op. cit.* (note 3), ch. 7.

69.

Larry O. Gostin , A Human Condition: The Mental Health Act from 1959 to 1975, Observations, Analysis and Proposals for Reform (London: Mind, 1975).

70.
Unsworth, *op. cit.* (note 17), 342–3.

71.
Gostin, *op. cit.* (note 69), 15.

72.
For example, *Co-ordination or Chaos? The Rundown of the Psychiatric Hospitals* (London: MIND, 1974); *MIND’s Evidence to the Committee of Enquiry into Special Education* (London: MIND, 1975); MIND, *The Mental Handicap Component in Social Work Training* (London: MIND, 1977).

73.
MIND, ‘MIND’s Evidence to the Peggy Jay Committee of Enquiry into the Care of the Mentally Handicapped’ (March 1976), 1. SA/MIN/B/80/10.

74.
MIND, *Services for Mentally Handicapped People: MIND’s Evidence to the Royal Commission on the National Health Service* (London: MIND, 1977).

75.

*Ibid.*, 18.

76.
For example, Editorial, ‘The Issues of Mental Handicap’, *MIND OUT*, 28 (1978), 2; Alison Wertheimer, ‘Researching into Mental Handicap’, *MIND OUT*, 40 (1980), 16–18: 17.

77.
Editorial, ‘The Issues of Mental Handicap’, *ibid.*, 2.

78.
MIND, *MIND’s Further Evidence to the Royal Commission on the NHS* (London: MIND, 1979), 10 (emphasis in original).

79.
MIND, *Services*, *op. cit.* (note 74), 27.

80.
MIND, *Services*, *op. cit.* (note 74), 4.

81.
MIND, *Services*, *op. cit.* (note 74), 28: 29.

82.
MIND, *Services*, *op. cit.* (note 74), 27; 36.

83.
MIND, *Services*, *op. cit.* (note 74), v: vi.

84.
MIND, *Services*, *op. cit.* (note 74), 29: 14.

85.
Indeed, this continued to leave people vulnerable to exploitation; see, for example, Granada TV’s 1978 *World in Action*which exposed the exploitation of learning disabled people in adult training centres: https://www.youtube.com/watch?v=MgfjUh2j2Mo (accessed 22 June 16).

86.

Kenneth Rawnsley , ‘Psychiatry in Jeopardy’, British Journal of Psychiatry, 145 (1984), 573–578: 577.650926610.1192/bjp.145.6.573

87.
In other words, that people will not need tailored support or specialist services. Alan Heaton-Ward, *Left Behind: A Study of Mental Handicap* (London: Routledge, 1978), 78–80.

88.
Report of the Committee of Enquiry into Mental Handicap Nursing and Care (Jay Committee) HMSO 1979 Cmnd 7468; MIND, *MIND’s Response to Jay*, (1979), 20.

89.
Valuing People: A New Strategy for Learning Disability for the 21st *Century*, Cm 5086 (London: Department of Health, 2001).

90.
Welshman and Walmsley (eds), *Community Care*, *op. cit.* (note 1), 223.

